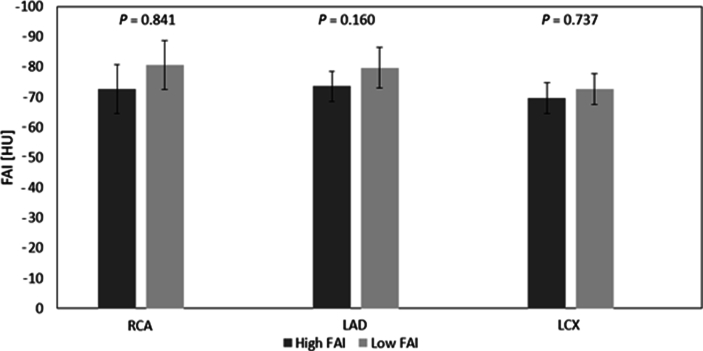# Perivascular fat attenuation index (FAI) on computed tomography coronary angiography reclassifies individual cardiovascular risk estimation

**DOI:** 10.1016/j.ijcrp.2024.200360

**Published:** 2024-12-18

**Authors:** Casper F. Coerkamp, Victor A. Verpalen, Remko S. Kuipers, Annet Driessen-Waaijer, Victor P.M. van der Hulst, Nils R. Planken, José P.S. Henriques, Robert K. Riezebos

**Affiliations:** a Department of Cardiology, Amsterdam University Medical Center, University of Amsterdam, Amsterdam Cardiovascular Sciences, Amsterdam, the Netherlands; b Department of Radiology and Nuclear Medicine, Amsterdam University Medical Center, University of Amsterdam, Amsterdam Cardiovascular Sciences, Amsterdam, the Netherlands; c Department of Cardiology, Zaans Medical Center, Zaandam, the Netherlands; d Department of Cardiology, OLVG Hospital, Amsterdam, the Netherlands; e Department of Cardiology, Isala Hospital, Zwolle, the Netherlands

**Keywords:** Coronary artery disease, Computed tomography coronary angiography, Coronary inflammation, Perivascular fat attenuation index

## Abstract

**Background:**

The perivascular fat attenuation index (FAI) detects and quantifies coronary inflammation by measuring phenotypic changes in perivascular adipose tissue by using computed tomography coronary angiography images.

**Aim:**

The primary objective of this study was to evaluate the reclassification of cardiovascular (CV) risk after incorporating perivascular FAI assessment in currently used risk score algorithms.

**Methods:**

This was a single-center, retrospective study of 200 patients with suspected coronary artery disease who underwent computed tomography coronary angiography in clinical practice between January 2022 and May 2022. From the patients who met the inclusion criteria, we included 50 patients with the highest CV risk according to the U-prevent calculator score to perform the perivascular FAI analysis. High perivascular FAI was defined as either a FAI-Score of ≥75th percentile in the left anterior descending artery or right coronary artery, or ≥95th percentile in the left circumflex artery.

**Results:**

In 62 % of the patients, there was a reclassification in CV risk after perivascular FAI assessment; individual risk was upgraded in 22 % of patients and in 40 % their risk was downgraded. The presence of any plaque (72.7 % vs. 94.1 %; *P* = 0.032) and the proportion of patients with moderate-to-high coronary artery calcium score (≥100 Agatston units) was higher in the high perivascular FAI group compared to the low FAI group (76.5 % vs. 36.4 %; *P* = 0.016). Major adverse cardiac and cerebrovascular events did not differ between both groups.

**Conclusion:**

The findings in this study suggest the potential valuable role of perivascular FAI assessment in individual CV risk prediction for patients with documented or suspected coronary artery disease.

## Highlights

• Perivascular FAI predicts cardiovascular risk in coronary artery disease patients.
• Perivascular FAI is a potential independent risk predictor.
• Perivascular FAI identifies patients with who need extensive secondary prevention.
• Risk reclassification leads to more targeted primary and secondary prevention therapy.

## Graphical abstract

**Figure:**
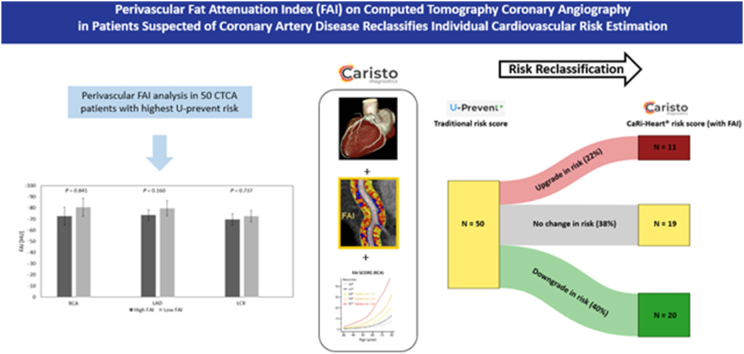

## **Abbreviations**

CAC: Coronary Artery Calcium Score
CAD: Coronary Artery disease
CAD-RADS: Coronary Artery Disease-Reporting and Data System
CVD: Cardiovascular Disease
CV: Cardiovascular
CTCA: Computed Tomography Coronary Angiography
FAI: Fat Attenuation Index
HU: Hounsfield Units
MACCE: Major Adverse Cardiac and Cerebrovascular Events
MI: Myocardial Infarction

## 1 Introduction

Computed tomography coronary angiography (CTCA) is often used as initial imaging modality in patients with stable chest pain suspected of coronary artery disease (CAD) as it increases the accuracy of CAD diagnosis [1,2]. CTCA aims to detect both non-obstructive and obstructive CAD, and allows for better targeting of preventive optimal medical therapy which is likely to reduce coronary death and non-fatal myocardial infarction (MI) [3]. In recent years, there has been a growing recognition of the various mechanisms underlying atherosclerotic disease, challenging a single description of the vulnerable atherosclerotic plaque which is prone to rupture [4]. Both inflammation and hyperlipidemia seem to contribute to the risk of future cardiovascular (CV) events [5,6]. There is increasing interest in markers of inflammation such as high-sensitivity C-reactive Protein to assess the residual CV risk [7]. This paradigm shift has resulted to the presumption of inflammation being the predisposing factor in atherosclerotic plaque formation. Previous randomized controlled trials have already confirmed that inflammation is a valid therapeutic target to reduce CV events [[8], [9], [10]]. Yet, these trials lack an individual coronary artery inflammation target selection and thus information of those patients who will benefit most from this anti-inflammatory therapy [[8], [9], [10]]. The perivascular fat attenuation index (FAI) detects and quantifies coronary inflammation by measuring phenotypic changes in perivascular adipose tissue by computed tomography post-processing imaging [11]. High perivascular FAI values identify high perivascular inflammation and have been associated with increased risk of cardiac death and non-fatal MI [12]. Therefore, we aimed to evaluate the impact of perivascular FAI assessment on individual CV risk reclassification when using a risk score based on traditional risk factors only [13]. We determined the proportion of patients that would be reclassified to either a higher or lower risk category by adding the perivascular FAI assessment to the currently used risk score algorithms. We also explored the potential clinical implications for patients with high perivascular FAI.

## 2 Methods

### 2.1 Study design and population

**Fig. 1:**
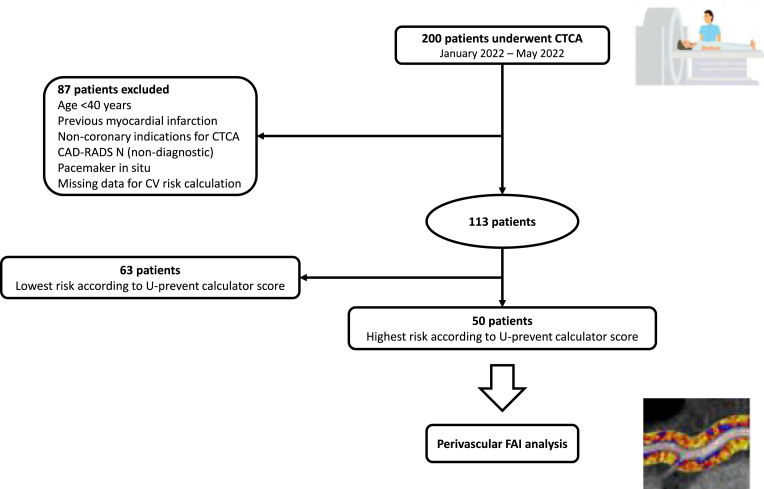
Flowchart demonstrating selection of the study population. The U-Prevent risk calculator predicts the 10-year CV mortality risk determined by the SCORE-2 risk chart for patients without previous CVD, for patients aged 70 and older without previous CVD the SCORE-OP-2 risk chart, for patients with previous CVD the SMART risk chart, and for patients with type 2 diabetes mellitus the ADVANCE risk chart. ADVANCE denotes Action in Diabetes and Vascular disease: pretax and diamicron-MR controlled evaluation, CAD-RADS Coronary Artery Disease-Reporting and Data System, CTCA computed tomography coronary angiography, CV cardiovascular, SCORE(-OP-)2 Systematic Coronary Risk Evaluation Older Persons-2, SMART Secondary Manifestations of Arterial disease.

CTCA was performed in 200 patients between January 2022 and May 2022 in the OLVG Hospital in the Netherlands. Patients were deemed suitable for inclusion if aged 40 years and above and if CTCA was indicated for suspected CAD. Patients were excluded if the CTCA images were of low-quality, indicating non-diagnostic scan according to the Classification of Coronary Artery Disease-Reporting and Data System (CAD-RADS) [14]. Patients were also excluded if CTCA was performed for non-coronary indications, if they had a previous MI or revascularization, receiving immunosuppressive therapy, had a pacemaker or in cases of missing data for calculation of the CV risk. From the remaining 113 patients who were eligible, we decided to include a total of 50 patients with the highest CV risk according to the U-prevent calculator score to perform the perivascular FAI analysis on (Fig. 1). The U-Prevent risk calculator predicts the 10-year CV mortality risk [13]. All data was collected manually from the electronic patient records of the OLVG Hospital and stored pseudo anonymized in the web-based electronic data capture system Castor. Fig. 3 was created by using the SankeyMATIC software. The study protocol is conform the ethical guidelines of the 1975 Declaration of Helsinki and was approved by the Scientific Research Advisory Committee of the OLVG hospital, which waived the need for informed consent.

### 2.2 Computed tomography coronary angiography and coronary artery calcium scoring

All patients were scanned using a 256-slice GE Revolution scanner. Oral or intravenous metoprolol were used for optimal heart rate and sublingual nitroglycerin for coronary vasodilation. The imaging protocol utilized a prospective electrocardiogram-gated sequential scanning technique. Omnipaque 300 mg l/ml was administered as the contrast agent, with contrast timing determined by the bolus-tracking methodology. The other CTCA technical parameters are depicted in Table S1. Coronary artery calcium score (CAC) scan was performed in all patients with calculation according to the Agatston-score [15]. The CTCA was assessed using the CAD-RADS method to classify coronary artery diameter stenosis [14].

### 2.3 Perivascular fat attenuation index

**Fig. 2:**
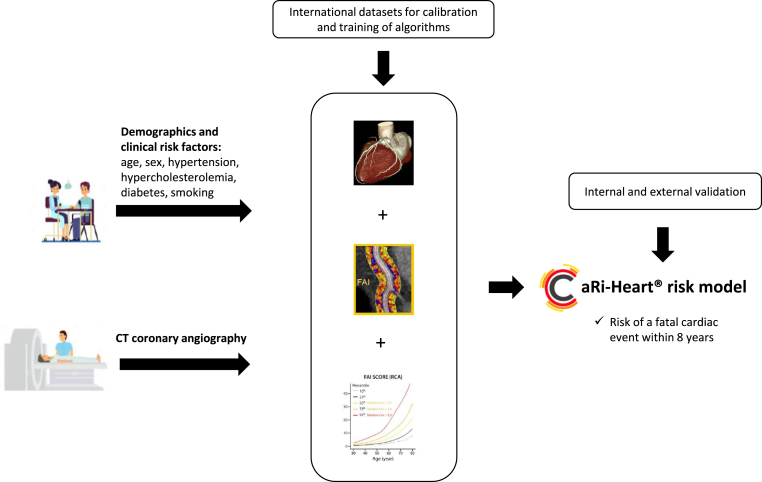
All computed tomography coronary angiography images and clinical risk factors are uploaded to the automated cloud-based medical device. The device performs automated segmentation of heart structures, identifies the perivascular adipose tissue, and calculates the perivascular FAI around each coronary vessel adjusted for technical and anatomical parameters, along with patient demographics. It also integrates a cardiac risk stratification tool for clinical decision making, the CaRi-Heart®. CAD denotes coronary artery disease, CT computed tomography, FAI fat attenuation index, RCA right coronary artery.

All CTCA images with their corresponding patient data were anonymized locally and uploaded to the CaRi-cloud of Caristo Diagnostics for the perivascular FAI analysis. The CaRi-Heart® Risk report including perivascular FAI-Scores, FAI-Score percentiles, and CaRi-Heart® 8-year risk prediction were returned after the analysis was performed (Fig. 2). To assess the perivascular FAI, the proximal 40-mm segment of the three coronary vessels was traced, defining the perivascular fat as adipose tissue within a radial distance from the outer vessel wall equivalent to the vessel's diameter. To mitigate effects of the aortic wall, the first proximal 10 mm of the RCA is excluded, and the subsequent 10–50 mm is analyzed. The left main was not analyzed due to its variable length. The perivascular FAI-Score was determined by quantifying the perivascular fat attenuation, adjusted for technical, anatomical and demographic factors, in the range of −190 to −30 Hounsfield Units (HU). The CaRi-Heart® 8-year risk model includes the FAI values, extent of CAD according to the Modified Duke coronary artery disease index [16], demographics, and clinical risk factors (diabetes, smoking, hyperlipidemia, and hypertension) [17]. The perivascular FAI analysis process is described in more detail previously [11,12].

**Fig. 3:**
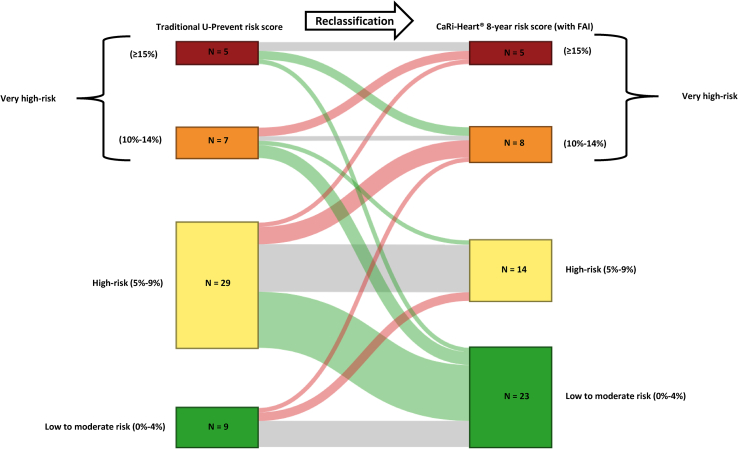
N = number of patients. Sankey diagram demonstrates cardiovascular (CV) risk reclassification of the risk models with (left) and without (right) addition of FAI. The proportion of patients without change (gray), reclassification to higher CV risk group (red), and reclassification to lower CV risk group (green) are shown. The U-Prevent risk calculator predicts the 10-year CV mortality risk determined by the SCORE-2 risk chart for patients without previous CVD, for patients aged 70 and older without previous CVD the SCORE-OP-2 risk chart, for patients with previous CVD the SMART risk chart, and for patients with type 2 diabetes mellitus the ADVANCE risk chart. FAI denotes fat attenuation index.

### 2.4 Outcomes

The primary objective of this study was to evaluate the reclassification of the estimated CV risk after incorporating perivascular FAI assessment. The key secondary outcome was to determine the perivascular FAI values and the distribution of perivascular FAI in a population of patients with suspected CAD who were selected based on the highest CV risk. Other outcomes consisted of major adverse cardiac and cerebrovascular events (MACCE) which was a composite of all-cause death, MI, ischemic stroke, transient ischemic attack, percutaneous coronary intervention, or coronary artery bypass grafting. The cut-off values for high perivascular FAI are according to previous studies and were determined based on percentiles, considering different age groups. High perivascular FAI was defined as either a perivascular FAI-Score of ≥75th percentile in the right coronary artery (RCA) or in the left anterior descending artery (LAD), or ≥95th percentile in the left circumflex artery (LCX) [17].

### 2.5 Statistical analysis

Normally distributed continuous variables were described as mean and standard deviations. Categorical data were described as numbers of patients with percentages. Continuous variables were compared across both groups using unpaired *T*-test or Mann-Whitney *U* test, whereas categorical variables were compared by Fisher's Exact test or the chi-square test. The CaRi-Heart® 8-year risk model was compared to the 10-year CV risk as calculated by U-Prevent risk calculator to better understand the additional value of FAI analysis in risk prediction. P-value of <0.05 was considered statistically significant. All statistical analyses were performed in SPSS Statistics version 28.0 (IBM, Armonk, New York).

## 3 Results

### 3.1 Baseline characteristics

Between January 2022 and May 2022, a total of 200 consecutive patients underwent CTCA in this single-center, observational study in the OLVG Hospital in the Netherlands. From these individuals, 113 patients were eligible for inclusion. We selected 50 patients with the highest 10-year predicted CV mortality risk, resulting in a mean risk of 9.0 ± 5.6 % across all included patients (Table 1). Of these 50 patients, 17 patients were classified into the high perivascular FAI group and 33 patients into the low perivascular FAI group. The mean age of all included patients was 62 ± 7.7 years and 60 % were female (Table 1). In the overall population, 46 % of the patients used statins during the CTCA scan. This did not differ between both groups (52.9 % vs. 42.4 %; *P* = 0.480). Pre-test probability for obstructive CAD and 10-year CV risk were similar between both groups (Table 1).

**Table 1:** Baseline characteristics of patients with high and low perivascular fat attenuation index values.

	All patients (n = 50)	High FAI-Score percentile (n = 17)	Low FAI-Score percentile (n = 33)	*P*-value
**Risk factors and lifestyle**				
Age (years)	62 ± 7.7 (50)	59.8 ± 8.5 (17)	63.2 ± 7.2 (33)	0.247
Male	40.0 % (20/50)	35.3 % (6/17)	42.4 % (14/33)	0.763
Hypertension	64.0 % (32/50)	70.6 % (12/17)	60.6 % (20/33)	0.486
Diabetes	16.0 % (8/50)	23.5 % (4/17)	12.1 % (4/33)	0.308
Dyslipidaemia	56.0 % (28/50)	58.8 % (10/17)	54.5 % (18/33)	0.773
Smoking status				
Current smoker	24.0 % (12/50)	29.4 % (5/17)	21.2 % (7/33)	0.763
Former smoker	22.0 % (11/50)	17.6 % (3/17)	24.4 % (8/33)	
Never smoker	54.0 % (27/50)	52.9 % (9/17)	54.5 % (18/33)	
Known family history of premature coronary disease	30.0 % (15/50)	35.3 % (6/17)	27.3 % (9/33)	0.558
Pre-test probability of CAD				
Basic model^a^	18.1 ± 15.4 (50)	15.1 ± 13.6 (17)	19.7 ± 16.3 (33)	0.329
Clinical model^b^	19.6 ± 16.1 (50)	17.9 ± 15.4 (17)	20.4 ± 16.4 (33)	0.611
Stroke	4.0 % (2/50)	5.9 % (1/17)	3.0 % (1/33)	0.635
**Clinical examination**				
Chest pain				
Typical	14.0 % (7/50)	11.8 % (2/17)	15.2 % (5/33)	0.944
Atypical	58.0 % (29/50)	58.8 % (10/17)	57.6 % (19/33)	
Non-anginal	28.0 % (14/50)	29.4 % (5/17)	27.3 % (9/33)	
Systolic blood pressure (mmHg)	158 ± 20.6 (50)	163 ± 18.2 (17)	155 ± 21.4 (33)	0.191
BMI (kg/m2)	29.6 ± 4.8 (50)	29.7 ± 5.4 (17)	29.6 ± 4.5 (33)	0.899
**Lipid lowering drug**				
Statin	46.0 % (23/50)	52.9 % (9/17)	42.4 % (14/33)	0.480
Ezetimibe	8.0 % (4/50)	5.9 % (1/17)	9.1 % (3/33)	0.580
**Laboratory parameters**				
(hs-)CRP (mg/l)	4.4 ± 5.0 (25)	2.1 ± 3.5 (7)	5.3 ± 5.2 (18)	0.148
eGFR (ml/min/1,73 m2)	79.0 ± 13.8 (48)	71.8 ± 17.7 (17)	83.0 ± 9.3 (31)	0.006
Cholesterol (mmol/l)	5.2 ± 1.4 (50)	5.1 ± 0.9 (17)	5.3 ± 1.5 (33)	0.604
LDL (mmol/l)	3.2 ± 1.2 (50)	3.0 ± 0.7 (17)	3.4 ± 1.3 (33)	0.255
HDL (mmol/l)	1.4 ± 0.5 (50)	1.4 ± 0.7 (17)	1.4 ± 0.4 (33)	0.814
Triglyceride (mmol/l)	1.9 ± 0.7 (16)	2.1 ± 0.4 (7)	1.8 ± 0.8 (9)	0.313
**10-year cardiovascular risk**				
U-prevent calculator score (%)^c^	9.0 ± 5.6 (50)	10.0 ± 8.0 (17)	8.6 ± 3.9 (33)	0.403

N = number of patients. Continuous variables were presented as mean ± standard deviation and compared across both groups by *t*-test with available data indicated in brackets. Categorical variables were expressed as counts and percentages and compared using χ2-test with the number of concerned patients/number of available data indicated in brackets. A FAI-Score of ≥75th percentile in the LAD or RCA, or ≥95th percentile in the LCX is considered to indicate increased risk and is therefore defined as a high FAI-Score percentile. ADVANCE denotes Action in Diabetes and Vascular disease: pretax and diamicron-MR controlled evaluation; BMI body mass index; CAD coronary artery disease; eGFR estimated glomerular filtration rate; FAI fat attenuation index; LAD left anterior descending artery; LCX left circumflex artery; LDL low-density lipoproteins; RCA right coronary artery; SCORE(-OP-)2 Systematic Coronary Risk Evaluation Older Persons-2; SMART Secondary Manifestations of Arterial disease.

a Pretest probability of obstructive CAD in patients with chest pain based on age, sex, and symptoms according to the basic predicition model developed by the CAD consortium.

b Pretest probability of obstructive CAD in patients with chest pain based on age, sex, symptoms, and cardiovascular risk factors including diabetes, hypertension, dyslipidemia, and smoking history according to the clinical predicition model developed by the CAD consortium.

c The U-Prevent risk calculator predicts the 10-year CV mortality risk determined by the SCORE-2 risk chart for patients without previous CVD, for patients aged 70 and older without previous CVD the SCORE-OP-2 risk chart, for patients with previous CVD the SMART risk chart, and for patients with type 2 diabetes mellitus the ADVANCE risk chart.

### 3.2 Perivascular FAI values

The mean perivascular FAI values did not differ between the high and low perivascular FAI group (Figure S1). The mean perivascular FAI-Score percentiles in the high and low perivascular FAI groups were 13.2 ± 9.4 (17) in RCA, 10.1 ± 5.0 (17) in LAD, 9.4 ± 4.1 (17) in LCX and 7.1 ± (33) in RCA, 7.8 ± 3.9 (33) in LAD, 10.4 ± 4.5 (32) in LCX, respectively. The overall mean CaRi-Heart 8-year risk was 9.5 ± 13.9 % (Table S2).

### 3.3 Coronary artery disease severity

Overall, 78 % of patients had presence of any plaque (CAD-RADS ≥1). When compared with the low perivascular FAI group, the presence of any plaque was higher in the high perivascular FAI group (72.7 % vs. 94.1 %; *P* = 0.032). In the overall population, 30 % of patients had obstructive CAD (CAD-RADS ≥3) (Table 2). The proportion of patients with obstructive CAD was similar between the high and low perivascular FAI groups. The proportion of patients with moderate-to-very high CAC (≥100 Agatston units) was greater in the high perivascular FAI group compared to the low perivascular FAI group (76.5 % vs. 36.4 %; *P* = 0.016) (Table 2).

**Table 2:** Coronary CT angiography findings of patients with high and low perivascular fat attenuation index values.

	All patients (n = 50)	High FAI-Score percentile (n = 17)	Low FAI-Score percentile (n = 33)	*P*-value
**Coronary artery calcium scoring (CAC)**				
Total CAC				
Zero (0)	22 % (11/50)	5.9 % (1/17)	30.3 % (10/33)	0.068
Minimal (1–9)	10 % (5/50)	5.9 % (1/17)	12.1 % (4/33)	
Low (10–99)	18 % (9/50)	11.8 % (2/17)	21.2 % (7/33)	
Moderate (100–399)	18 % (9/50)	35.3 % (6/17)	9.1 % (3/33)	
High (400–999)	24 % (12/50)	35.3 % (6/17)	18.2 % (6/33	
Very high (≥1000)	8 % (4/50)	5.9 % (1/17)	9.1 % (3/33)	
Total CAC				
<100	50 % (25/50)	23.5 % (4/17)	63.6 % (21/33)	0.016
≥100	50 % (25/50)	76.5 % (13/17)	36.4 % (12/33)	
(mesa) percentile	55.7 ± 35.9 (50)	76.1 ± 23.8 (17)	45.2 ± 36.9 (33)	0.003
**Angiographic scores**				
Number of vessels with lesions				
0 vessels	22 % (11/50)	5.9 % (1/17)	30.3 % (10/33)	0.136
1–2 vessels	46 % (23/50)	52.9 % (9/17)	42.4 % (14/33)	
3 vessels	32 % (16/50)	41.2 % (7/17)	27.3 % (9/33)	
Overall CAD-RADS				
0 (0 %)	22 % (11/50)	5.9 % (1/17)	30.3 % (10/33)	0.212
1 (1–24 %)	26 % (13/50)	23.5 % (4/17)	27.3 % (9/33)	
2 (25–49 %)	22 % (11/50)	29.4 % (5/17)	18.2 % (6/33)	
3 (50–69 %)	10 % (5/50)	17.6 % (3/17)	6.1 % (2/33)	
4a (70–99 %)	16 % (8/50)	23.5 % (4/17)	12.1 % (4/33)	
4b (LM ≥ 50 % of 3-VD ≥70 %)	2 % (1/50)	0 % (0/17)	3.0 % (1/33)	
5 (100 %)	2 % (1/50)	0 % (0/17)	3.0 % (1/33)^a^	
Overall CAD-RADS				
<3	70 % (35/50)	58.8 % (10/17)	75.8 % (25/33)	0.216
≥3	30 % (15/50)	41.2 % (7/17)	24.2 % (8/33)	
Presence of plaque (CAD-RADS ≥1)	78 % (39/50)	94.1 % (16/17)	72.7 % (23/33)	0.032
Plaque burden				
P1 (mild)	28 % (14/50)	29.4 % (5/17)	27.3 % (9/33)	0.289
P2 (moderate)	16 % (8/50)	17.6 % (3/17)	15.2 % (5/33)	
P3 (severe)	30 % (15/50)	41.2 % (7/17)	24.2 % (8/33)	
P4 (extensive amount)	4 % (2/50)	5.9 % (1/17)	3.0 % (1/33)	
Plaque burden (≥P3)	34 % (17/50)	47.0 % (8/17)	27.3 % (9/33)	0.162

N = number of patients.

a One patient had a chronic total occlusion (CTO) of the proximal LCX, as a result of which it is not possible to calculate the FAI-Score. Continuous variables were presented as mean ± standard deviation and compared across both groups by *t*-test with available data indicated in brackets. Categorical variables were expressed as counts and percentages and compared using χ2-test with the number of concerned patients/number of available data indicated in brackets. A FAI-score of ≥75th percentile in the LAD or RCA, or ≥95th percentile in the LCX is considered to indicate increased risk and is therefore defined as a high FAI-Score percentile. CAD-RADS denotes Coronary Artery Disease-Reporting and Data System; CT computed tomography; FAI fat attenuation index; LAD left anterior descending artery; LCX left circumflex artery; LM left main; RCA right coronary artery; VD vessel disease.

### 3.4 Risk reclassification

The mean 10-year CV risk, as calculated by the U-prevent risk calculator, was 9.0 ± 5.6 %, while the mean CaRi-Heart® 8-year risk (with addition of perivascular FAI) was 9.5 ± 13.9 %. When comparing CV risk calculated by the U-prevent risk calculator score with that calculated by the CaRi-Heart® risk model for high and low perivascular FAI, a risk reclassification was observed, with the mean risk changing from 10.0 ± 8.0 % to 16.5 ± 21.6 % (high perivascular FAI) and from 8.6 ± 3.9 % to 5.9 ± 4.8 % (low perivascular FAI), respectively. This reclassification of CV risk is further illustrated in Fig. 3. The CV risk was classified in five different CV risk groups according to European Society of Cardiology guidelines [18]. The reclassifications between the U-prevent risk calculator score and the CaRi-Heart® Risk model were more often downgrades to a lower CV risk group (40 % [20/50]) compared to upgrades to a higher CV risk group (22 % [11/50]) according to the five different CV risk groups. In only 38 % (19/50) of the patients there was no change in CV risk group.

### 3.5 Major adverse cardiac and cerebrovascular events

The mean follow-up duration is 2.1 ± 0.1 years. In total, 14 % (7/50) patients developed MACCE. The prevalence of MACCE was 17.6 % in the high perivascular FAI group compared with 12.1 % in the low perivascular FAI group (*P* = 0.677) (Table S3). None of the patients died during the follow-up period. The proportion of patients who experienced MI was similar between both groups (5.9 % vs. 6.1 %, *P* = 0.980). Compared with the low perivascular FAI group, the proportion of patients who underwent percutaneous coronary intervention was higher in the high perivascular FAI group, however this difference was not statistically significant (17.6 % vs. 6.1 %; *P* = 0.209) (Table S3).

## 4 Discussion

This study provides new insights on how FAI changes individual CV risk prediction, with a reclassification in 62 % of the patients in the study cohort. It adds to evidence from the Cardiovascular Risk Prediction using Computed Tomography (CRISP-CT) study, which demonstrated a correlation between high perivascular FAI and cardiac mortality and nonfatal MI in both derivation and validation cohorts [12]. This is likely caused by coronary inflammation which can be detected by FAI and as such identifies vulnerable plaques which are prone to rupture. Overall MACCE was low in our study, as in previous studies with similar patient populations [3]. However, our study showed that perivascular FAI analysis led to reclassification of CV risk in a staggering 62 % (31/50) of the patients, enabling the identification of patients who are likely to benefit from more extensive secondary prevention or alternatively to reclassification towards lower CV risk. Our study supports the findings of the recent published Oxford Risk Factors And Non-invasive imaging (ORFAN) study, which demonstrated the inflammatory risk beyond the traditional risk factor-based risk calculators of 3393 individuals, showing that perivascular FAI leads to the reclassification of CV risk [19]. In our study, the results also indicate that the risk of high perivascular FAI may be independent of age, sex and traditional risk factors for CAD such as hypertension, diabetes, hypercholesterolemia, obesity, and smoking. We found that patients with high perivascular FAI potentially have a higher prevalence of CAD and a greater burden of coronary calcification compared to patients with low perivascular FAI. It has been shown previously that statin therapy reduces perivascular FAI while also increasing the total plaque volume and the volume of the calcified component in non-calcified and mixed plaques [20]. In our study, 46 % of the patients used statins during the CTCA scan, which corresponds to the statin use observed in the CRISP-CT study, with 35 % in the derivation cohort and 40 % in the validation cohort [12].

This perivascular inflammation leads to cellular modifications in the coronary arteries that can occur before they are even detectable on the current diagnostic imaging tests [21]. Currently there are still patients who suffer from MI or cardiac death as first manifestation or that have been missed by conventional diagnostic tools [19]. The perivascular FAI analysis can be performed on routine CTCA, allowing cardiologists to identify those patients who are at the highest risk for future cardiac events [12].

### 4.1 Study limitations

This study has several limitations. First, it compromises a relatively small patient cohort (N = 50) from a single-center, limiting its representativeness and reducing its ability to determine a linear or other type of relationship between high perivascular FAI and CAD burden. Secondly, as this is cross-sectional cohort study, this study has inherent limitations on drawing conclusions regarding causal associations between perivascular FAI and CAD. Thirdly, the limited occurrence of MACCE reported in this study precludes conclusions of the prognostic value in this cohort. Fourthly, the left main and the first proximal 10 mm of the RCA were excluded in the FAI analysis. Finally, although we have data on statin usage during CTCA scans, data on duration of statin treatment is missing.

### 4.2 Clinical implications

Perivascular FAI assessment might contribute to the identification of patients with CAD who may benefit from more extensive secondary prevention. In this study, however, perivascular FAI assessment most frequently led to the downgrading of patients to a lower CV risk group, possibly due to selecting patients with the highest CV risk according to the U-prevent calculator score. This reclassification could potentially result in more restricted primary and secondary prevention therapy. The findings in this study underscore the valuable role of perivascular FAI assessment in CV risk prediction for patients with documented or suspected CAD. Furthermore, the results of this study also indicate that perivascular FAI is a potential independent risk predictor that can complement existing risk predictors. Further research is awaited to investigate the effects of anti-inflammatory drugs such as colchicine (NCT05347316, NCT06083337) on perivascular FAI, plaque progression and its impact on CV events.

## CRediT authorship contribution statement

**Casper F. Coerkamp:** Writing – review & editing, Writing – original draft, Visualization, Validation, Software, Project administration, Methodology, Investigation, Formal analysis, Conceptualization. **Victor A. Verpalen:** Writing – review & editing. **Remko S. Kuipers:** Writing – review & editing, Conceptualization. **Annet Driessen-Waaijer:** Writing – review & editing. **Victor P.M. van der Hulst:** Writing – review & editing. **Nils R. Planken:** Writing – review & editing, Methodology. **José P.S. Henriques:** Writing – review & editing, Writing – original draft, Methodology, Conceptualization. **Robert K. Riezebos:** Writing – review & editing, Writing – original draft, Supervision, Methodology.

## Funding

Caristo Diagnostics performed the perivascular fat attenuation analysis in kind.Structured

## Declaration of competing interest

We have no conflict of interest to declare.

## Figures and Tables

**figs1:**