# Supplementary material for: Perivascular fat attenuation index (FAI) on computed tomography coronary angiography reclassifies individual cardiovascular risk estimation
Source: Int J Cardiol Cardiovasc Risk Prev. 2024 Dec 18;24:200360. doi: 10.1016/j.ijcrp.2024.200360 (PMC11732156; doi:10.1016/j.ijcrp.2024.200360)
Supplement: Multimedia component 1 [file mmc1.docx]

**Table S1: Coronary CT angiography technical parameters**

|  | All patients  (n=50) | High FAI-Score percentile  (n=17) | Low FAI-Score percentile  (n=33) | *P*-value |
| --- | --- | --- | --- | --- |
| CTCA acquisition parameters |  |  |  |  |
| Heart rate [beats/min] | 59 ± 8 (50) | 60 ± 8 (17) | 58 ± 7 (33) | 0.524 |
| Tube voltage [kVp] |  |  |  |  |
| 100 kVp | 48% (24/50) | 64.7% (11/17) | 39.4% (13/33) | 0.090 |
| 120 kVp | 52% (26/50) | 35.3% (6/17) | 60.6% (20/33) |  |
| Tube current [mAs] | 558 ± 128 (50) | 536 ± 132 (17) | 570 ± 126 (33) | 0.372 |
| Contrast volume [ml] | 92 ± 9 (50) | 91 ± 10 (17) | 93 ± 9 (33) | 0.593 |
| Radiation dose of CTCA [DLP] | 245 ± 110 (50) | 233 ± 120 (17) | 250 ± 107 (33) | 0.609 |
| Total effective dose [mSv] | 4.1 ± 1.7 (50) | 3.9 ± 1.8 (17) | 4.1 ± 1.7 (33) | 0.756 |
| Attenuation parameters |  |  |  |  |
| Aorta [HU] | 542 ± 108 (50) | 547 ± 112 (17) | 540 ± 107 (33) | 0.850 |
| Aorta SD [HU] | 29 ± 6 (50) | 28 ± 6 (17) | 24 ± 6 (33) | 0.034 |
| RCA [HU] | 483 ± 98 (50) | 467 ± 110 (17) | 491 ± 93 (33) | 0.420 |
| RCA SD [HU] | 46 ± 17 (50) | 43 ± 18 (17) | 47 ± 17 (33) | 0.462 |
| LAD [HU] | 520 ± 141 (50) | 544 ± 169 (17) | 507 ± 125 (33) | 0.378 |
| LAD SD [HU] | 73 ± 50 (50) | 89 ± 71 (17) | 65 ± 33 (33) | 0.211 |

N = number of patients. Continuous variables were presented as mean ± standard deviation and compared across both groups by t-test with available data indicated in brackets. Categorical variables were expressed as counts and percentages and compared using χ2-test with the number of concerned patients/number of available data indicated in brackets. A FAI-Score of ≥75^th^ percentile in the LAD or RCA, or ≥95^th^ percentile in the LCX is considered to indicate increased risk and is therefore defined as a high FAI-Score percentile. CTCA denotes computed tomography coronary angiography; FAI fat attenuation index; HU Hounsfield units; LAD left anterior descending artery; LCX left circumflex artery; RCA right coronary artery; SD standard deviation.

**Table S2: Perivascular fat attenuation index values**

|  | All patients  (n=50) | High FAI-Score percentile (n = 17) | Low FAI-Score percentile  (N = 33) | *P*-value |
| --- | --- | --- | --- | --- |
| FAI [HU] |  |  |  |  |
| RCA | -77.8 ± 9.0 (50) | -72.2 ± 8.2 (17) | -80.7 ± 8.1 (33) | 0.841 |
| LAD | -77.7 ± 6.8 (50) | -73.6 ± 5.0 (17) | -79.8 ± 6.7 (33) | 0.160 |
| LCX | -71.7 ± 5.2 (49)^1^ | -69.8 ± 5.1 (17) | -72.7 ± 5.0 (32)^1^ | 0.737 |
| Total (n=149 vessels) | -75.7 ± 7.7 (149)^1^ | - | - | - |
| FAI-Score |  |  |  |  |
| RCA | 9.2 ± 7.0 (50) | 13.2 ± 9.4 (17) | 7.1 ± 4.3 (33) | 0.011 |
| LAD | 8.6 ± 4.4 (50) | 10.1 ± 5.0 (17) | 7.8 ± 3.9 (33) | 0.189 |
| LCX | 10.1 ± 4.4 (49)^1^ | 9.4 ± 4.1 (17) | 10.4 ± 4.5 (32)^1^ | 0.349 |
| FAI-Score percentile |  |  |  |  |
| RCA | 54.8 ± 26.9 (50) | 76.1 ± 21.2 (17) | 43.9 ± 22.8 (33) | 0.225 |
| LAD | 56.8 ± 23.9 (50) | 76.4 ± 18.3 (17) | 46.7 ± 20.0 (33) | 0.292 |
| LCX | 72.3 ± 21.9 (49)^1^ | 80.2 ± 20.7 (17) | 68.1 ± 21.6 (33) | 0.213 |
| Risk prediction |  |  |  |  |
| CaRi-Heart Risk (8 year risk) | 9.5 ± 13.9 (50) | 16.5 ± 21.6 (17) | 5.9 ± 4.8 (33) | <0.001 |

N = number of patients. ^1^One patient had a chronic total occlusion (CTO) of the proximal LCX, making it not possible to calculate the FAI. Continuous variables were presented as mean ± standard deviation and compared across both groups by t-test with available data indicated in brackets. A FAI-Score of ≥75^th^ percentile in the LAD or RCA, or ≥95^th^ percentile in the LCX is considered to indicate increased risk and is therefore defined as a high FAI-Score percentile. CTO denotes chronic total occlusion; FAI fat attenuation index; HU Hounsfield Units; LAD left anterior descending artery; LCX left circumflex artery; RCA right coronary artery.

**Table S3: Major adverse cardiac and cerebrovascular events**

|  | High FAI percentile  (n=17) | Low FAI percentile  (n=33) | P-value |
| --- | --- | --- | --- |
| MACCE | 17.6% (3/17) | 12.1% (4/33) | 0.677 |
| All-cause death | 0% (0/17) | 0% (0/33) | NA |
| Myocardial infarction | 5.9% (1/17) | 6.1% (2/33) | 0.980 |
| TIA or ischemic stroke | 0% (0/17) | 3.0% (1/33) | 0.660 |
| Revascularisation | | | |
| PCI | 17.6% (3/17) | 6.1% (2/33) | 0.209 |
| CABG | 0% (0/17) | 0% (0/33) | NA |

N = number of patients. Categorical variables were expressed as counts and percentages and compared using χ2-test with the number of concerned patients/number of available data indicated in brackets. A FAI score of ≥75 percentile in the LAD or RCA, or ≥95 percentile in the LCX is considered to indicate increased risk and is therefore defined as a high FAI percentile. CABG denotes coronary artery bypass grafting; FAI fat attenuation index; MACCE major adverse cardiac and cerebrovascular events; PCI percutaneous coronary intervention; TIA transient ischemic attack.
